# Patient and Physician Preferences for Regimen Attributes for the Treatment of HIV in the United States and Canada

**DOI:** 10.3390/jpm12030334

**Published:** 2022-02-23

**Authors:** Heather Gelhorn, Cindy Garris, Erin Arthurs, Frank Spinelli, Katelyn Cutts, Gin Nie Chua, Hannah Collacott, Bertrand Lebouché, Erik Lowman, Howard Rice, Sebastian Heidenreich

**Affiliations:** 1 Evidera, Bethesda, MD 20814, USA; heather.gelhorn@evidera.com (H.G.); katelyn.cutts@evidera.com (K.C.); 2 US Health Outcomes, ViiV Healthcare, Research Triangle Park, NC 27709, USA; 3 GlaxoSmithKline, Mississauga, ON L5N 6L4, Canada; erin.x.arthurs@gsk.com; 4 North American Medical Affairs, ViiV Healthcare, Research Triangle Park, NC 27709, USA; frank.a.spinelli@viivhealthcare.com; 5 Evidera, London W6 8BJ, UK; ginniechua@gmail.com (G.N.C.); hannah.collacott@evidera.com (H.C.); sebastian.heidenreich@evidera.com (S.H.); 6 Centre for Outcomes Research & Evaluation, Research Institute of the McGill University Health Centre, Montreal, QC H3H 2R9, Canada; bertrand.lebouche@mcgill.ca; 7 Chronic Viral Illness Service, Division of Infectious Diseases, Department of Medicine, McGill University Health Centre, Montreal, QC H4A 3J1, Canada; 8 Department of Family Medicine, Faculty of Medicine, McGill University, Montreal, QC H3S 1Z1, Canada; 9 Midland Medical Center, Oakland Park, FL 33334, USA; elowman@midlandmed.com; 10 Rice Medical Group, Mountain View, CA 94040, USA; drrice@drrice.com

**Keywords:** antiretroviral therapy, discrete choice experiment (DCE), HIV, long-acting injectable (LAI), patient preference, physician preference

## Abstract

A long-acting injectable (LAI) antiretroviral therapy (ART) regimen is now available as a treatment option for virologically suppressed adults with HIV-1. This study assessed preference for a LAI regimen using an online survey of virally suppressed people living with HIV (PLWH) and physicians treating HIV in the US and Canada. Preference was elicited in a discrete choice experiment (DCE) with three choice options (switch to a LAI regimen, switch to another daily oral ART regimen, or stay on their current daily oral ART regimen) and four treatment attributes. A total of 553 PLWH and 450 physicians completed the survey. From the DCE results, 59% of PLWH were predicted to prefer a LAI over an alternative oral ART or staying on their current oral treatment, and 55–66% of physicians were predicted to recommend LAI for PLWH, depending on the treatment challenge scenario presented. PLWH indicated LAI would remove daily reminders of HIV (75%) and reduce feelings of being stigmatized (68%). A majority of PLWH and physicians preferred a LAI over oral ART to overcome treatment challenges such as daily pill burden and adherence. These benefits of LAI ART along with preferences of PLWH and physicians can help to inform ART choice.

## 1. Introduction

Advances in antiretroviral therapy (ART) for the treatment of human immunodeficiency virus (HIV) have significantly improved outcomes for people living with HIV (PLWH), with projected life-expectancy for PLWH approaching 90% of that of the general population in some countries [1]. As such, HIV is now managed as a chronic disease, increasingly affecting an aging population [2,3,4,5], which has revealed a new set of treatment challenges. Until recently, the standard of care for HIV therapy has been a once daily oral combination ART regimen, which requires continuous high adherence to maintain viral suppression [6].

Social stigma, socioeconomic status, poor health literacy, drug abuse, ART-associated side-effects, cumulative drug toxicity, and pill-burden resulting from taking daily ART pills have all been associated with suboptimal adherence [7,8,9]. In turn, poor adherence to ART among PLWH is associated with reduced viral suppression, increased drug-resistance and more hospitalizations compared with adherent PLWH [10,11]. Thus, it is important to optimize ART and provide additional treatment options that better address patients’ needs.

One strategy to enable maintenance of optimal adherence to ART over the long-term is to provide alternative dosing/delivery methods that negate the need to take a daily pill or pills. The ATLAS (NCT02951052) and FLAIR (NCT02938520) Phase 3 clinical trials demonstrated that the long-acting regimen of cabotegravir + rilpivirine, given as a monthly injection, had similar efficacy to current daily oral ART [12]. Further, the ATLAS-2M Phase 3 study (NCT03299049) demonstrated that cabotegravir + rilpivirine long-acting administered every two months had similar efficacy to monthly administration [13]. Patients receiving the long-acting injectable (LAI) reported higher levels of treatment satisfaction and preference over daily oral ART [12], as well as a higher preference for dosing every two months over monthly dosing [14]. This LAI treatment option of cabotegravir and rilpivirine, indicated as a complete regimen for the treatment of HIV in virologically suppressed adults, has received regulatory approval in the US [15] and Canada [16] for both once monthly and every two months dosing.

Participants in the clinical trials were enrolled based on their willingness to switch to a LAI and as such, little is known about the attitudes of the wider PLWH community and their willingness to take a LAI instead of an oral ART. Moreover, it is important to understand the perspective of physicians treating PLWH. This study assessed PLWH’s and physicians’ preferences for switching from current daily oral ART to a LAI ART, along with participant satisfaction and unmet needs with current ART.

## 2. Materials and Methods

### 2.1. Study Design

The study involved an online survey administered to adult PLWH and physicians treating HIV, consisting of an introduction, participant information and four sections. The survey was developed based on input from previous qualitative research, clinical experts, and pilot interviews with PLWH and physicians. Further details on the survey development are described below. In the participant information section of the survey, PLWH and physicians were provided with an overview of LAI ART, including administration (i.e., two injections administered in the gluteal muscle by a healthcare professional at the same visit, either monthly or every two months), and assumptions around efficacy (i.e., as effective as oral HIV treatment for maintaining viral suppression) and injection site reactions (i.e., most commonly reported as mild pain lasting for 3 days or less, with <1% of patients discontinuing due to injection site reactions). Section 1 of the survey included questions about current oral ART treatment satisfaction and treatment challenges. Section 2 presented participants with a discrete choice experiment (DCE), which is an established quantitative method to elicit treatment preferences [17,18]. Section 3 and Section 4 included debriefing about responses in the DCE, and clinical and sociodemographic questions, respectively. Spanish and French versions of the surveys were available. The self-completed online survey was pre-tested in qualitative and quantitative pilots before proceeding to the main data collection. The study protocol was approved by Advarra (Pro00030831) institutional review board, and all participants provided informed consent.

### 2.2. Study Population

To be eligible for participation in this study, PLWH had to be at least 18 years old, currently residing in the US or Canada, self-report having HIV and taking oral ART for at least 3 months at the time of consent, and be currently virally suppressed (undefined threshold), as the indication of the LAI injectable is for virally suppressed PLWH. Physicians had to be medically trained with at least 2 years of experience treating PLWH and reside in the US or Canada. All participants in the study were recruited from online access panels or databases. Eligibility was assessed using a series of self-completed online screening questions. Patient and provider participants were compensated a modest amount for their time to complete the survey.

### 2.3. Satisfaction and Stigma Questions

In the online survey, PLWH and physicians were asked to rate their level of satisfaction with current ART treatments, with the range of responses from (1) totally unsatisfied to (7) totally satisfied. They were also asked to select reasons for dissatisfaction if not totally satisfied. PLWH also answered questions relating to the stigma of living with HIV; these questions were developed by the researchers specifically for this survey. An overall stigma score was calculated for each PLWH participant based on their level of agreement with statements such as worrying about people finding out about their HIV status, feeling ashamed, and feeling hindered about being intimate with new partners due to their HIV. The total score (7 being lowest level of stigma; 28 being highest level of stigma) was generated based on the sum of the responses across all the stigma items. Scores were dichotomized as high or low, with the mean stigma score used as a cut-off point.

### 2.4. Discrete Choice Experiment

To inform the design of the DCE, recently published preference studies that discussed the risks and benefits of existing HIV treatment, published clinical evidence and qualitative research were reviewed to help identify treatment attributes and levels that could potentially distinguish between different ART regimes [19,20,21,22]. Potential attributes and commonly observed treatment challenges were discussed in two workshops with three HIV clinical experts (EL, HR, BL) and tested for completeness, relevance, and clarity in subsequent qualitative pilot interviews with PLWH and physicians.

Separate D-efficient DCE designs with 48 choice tasks were generated for PLWH and physicians and split into four blocks for PLWH and three blocks for physicians. Each block was randomly assigned to a participant, such that PLWH completed 12 and physicians completed 16 experimental choice tasks. The ordering of choice tasks and alternatives was randomized between participants to avoid left-right bias as well as learning and fatigue effects [23]. To assess the internal validity of answers and data quality, the sixth choice set as seen by the participant was repeated at the end of the survey to explore choice stability [24]. However, only the experimental choice tasks from the D-efficient design were used for model estimation. Small directional priors were used in the development of the D-efficient design.

**Figure 1:**
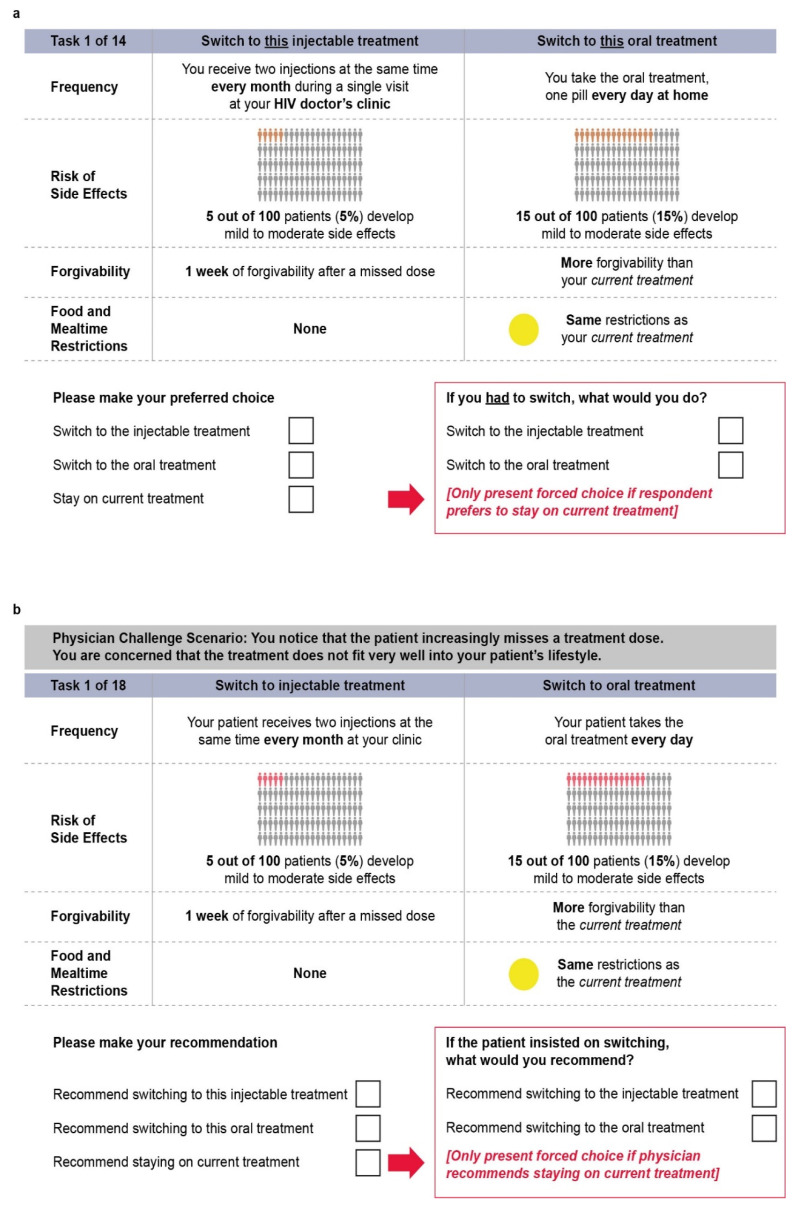
(**a**) Example PLWH choice task and (**b**) example physician choice task. If PLWH or physicians chose to stay on their current treatment or recommend that PLWH remain on current treatment, they were prompted with a forced choice task asking them to choose between switching to the LAI or oral treatment, shown in the red box. HIV = human immunodeficiency virus; PLWH = people living with HIV.

Within each DCE choice task, participants were asked to choose between switching to a LAI treatment administered every one or two months, switching to an alternative oral ART, or staying with their current oral ART. Participants were instructed to assume that the treatment choices offered differed only on the presented treatment aspects, all treatments would maintain an undetectable viral load, and the cost of the treatment choices was the same as what they were currently paying for their HIV treatment. The three choice alternatives were made up of four attributes (Table 1) with levels varied according to the generated experimental design. The attributes were: (1) dosing frequency, (2) risk of side effects, (3) forgivability, and (4) food/mealtime restrictions. Example choice tasks are presented in Figure 1a,b, for patients and physicians, respectively.

The overall choice setting for PLWH was based on the most relevant treatment challenge that PLWH selected from a prespecified set of scenarios that were developed during the workshops with the HIV clinical experts and validated during the pilot interviews (Table 2). PLWH were asked to imagine they were discussing options with their physician to potentially overcome their selected treatment challenge. Similarly, physicians were asked to imagine a patient requesting advice about a specific HIV treatment challenge. The treatment challenges presented to physicians were integrated into the experimental design as attribute levels of the current ART and matched the challenges presented to PLWH, but with the “no challenge” scenario being excluded to ensure a credible clinical setting (Table 2). The treatment challenge scenarios that physicians were asked to consider varied between choice tasks.

### 2.5. Demographics and Treatment Characteristics

After completing the DCE tasks, PLWH were presented questions about their demographic and clinical characteristics, including age, gender, race, sexual orientation, time since HIV diagnosis, time on current HIV therapy, adherence to HIV therapy, and frequency of medical visits to their providers. Physicians provided information related to their years of clinical experience, including time treating HIV, their specialty, and the type of clinic they work in. All information provided by participants was self-reported and not independently verified.

### 2.6. Pilot Testing

Qualitative pilot interviews were conducted with 10 PLWH and 10 physicians, with equal numbers of participants from both Canada and the US, to assess the clarity and comprehension of the DCE and overall survey. The pre-testing interviews also assessed the relevance as well as completeness of the included attributes and presented treatment challenges. For this purpose, participants were asked to complete the online surveys while thinking aloud. Interviewers observed participants and probed carefully to understand their comprehension of the survey questions. Minor revisions were made to the survey as a result of the pilot interviews, such as wording changes and renaming of attribute labels. Finally, a quantitative pilot was conducted with a further 50 PLWH and 50 physicians from the US. No revisions were made following the quantitative pilot.

### 2.7. Data Analysis

Descriptive statistics (i.e., means, SDs, ranges) were computed for data relating to sociodemographic and clinical information, health literacy, treatment satisfaction and stigma.

Multinomial logit and mixed logit models were used to analyze the DCE data. The multinomial logit was used to analyze the overall sample and the mixed logit was used for subgroup analyses. These analyses followed random utility theory (RUT), by assuming that in each choice task *t*, every participant *n*, chose the DCE alternative with the highest overall utility (i.e., treatment satisfaction). A separate utility function was defined for each of the three choice alternatives in the DCE:

(1) $$\begin{array}{c} u_{nt}^{\mathrm{LAI}}=LAI_{n}+\beta_{1\;}\mathrm{Frequency}\_2{\;\mathrm{months}}_{nt}+\beta_{2}\mathrm{Risk}\_15_{nt}^{\mathrm{LAI}}+\beta_{3}\mathrm{Risk}\_5_{nt}^{\mathrm{LAI}}+\\ \beta_{4}\mathrm{Forgive}\_2_{jnt}+\beta_{5}\mathrm{Forgive}\_3_{jnt}+\varepsilon_{nt}^{\mathrm{LAI}}\; \end{array}$$

(2) $$\begin{array}{ll} u_{nt}^{\mathrm{oral}}=oral_{n}+ & \beta_{2}\mathrm{Risk}\_15_{nt}^{\mathrm{oral}}+\beta_{3}\mathrm{Risk}\_5_{nt}^{\mathrm{oral}}+\beta_{6\;}\mathrm{forgive}\_{\mathrm{same}}_{jnt}\\ & +\beta_{7\;}\mathrm{forgive}\_{\mathrm{more}}_{jnt}+\beta_{8\;}\mathrm{restrict}\_{\mathrm{same}}_{jnt}\\ & +\beta_{9\;}\mathrm{restrict}\_{\mathrm{fewer}}_{jnt}+\varepsilon_{nt}^{\mathrm{oral}}\; \end{array}$$

(3) $$\begin{array}{c} u_{nt}^{\mathrm{current}}=1_{n}^{\mathrm{phy}}(\beta_{10}{\mathrm{Adhere}}_{jnt}+\beta_{11}{\mathrm{Worry}}_{jnt}+\beta_{12}\mathrm{Side}\_{\mathrm{effect}}_{jnt}+\\ \beta_{13}{\mathrm{Disclosure}}_{jnt}\;)+\varepsilon_{nt}^{\mathrm{current}}\; \end{array}$$

The variable names are defined in Table 1 and Table 2. The $\beta_{1}$ to $\beta_{13}$ parameters are referred to as marginal utilities and relate the attribute levels to the choices that participants made. Attributes were effects coded, such that a positive marginal utility indicated that an attribute level was preferred relative to the other levels of the same attribute. Marginal utilities of the reference level were recovered from the estimates and standard errors obtained using the delta method. The utility of the current ART was fixed to zero for PLWH but depended on the different switching scenarios (i.e., $\beta_{10}$ to $\beta_{13}$) for physicians. The alternative specific constants $LAI_{n}$ and $oral_{n}$ were included to capture participants’ switching tendency. For example, a positive $LAI_{n}$ would indicate that participants (ceteris paribus) tend to switch to a LAI ART instead of staying on their current ART. The utility functions were estimated as mixed logit models using STATA MP 15^1^ and model fit was assessed using the Adjusted McFadden R^2^.

Two additional outputs were obtained to facilitate the interpretation of the DCE results: First, switching probabilities were calculated for monthly and every two months LAI versus current ART and switching to the best-case oral alternative (i.e., more forgivability and fewer food and mealtime restrictions than the PLWH’s current oral treatment). For this analysis, the oral ART and LAI ART alternatives were both fixed to have a 5% risk of side effects as that is the lowest side effect level. Second, the most likely alternative $LAI_{n}$ and $oral_{n}$ parameters were obtained for each PLWH in the sample. The share of PLWH with a positive $LAI_{n}$ and a negative $oral_{n}$ were obtained to understand how many participants showed patterns of preferences which suggested that they might perceive that currently available oral ART alternatives may not overcome treatment challenges, while a LAI alternative potentially could.

To explore heterogeneity in preferences, PLWH’s sociodemographic and clinical characteristics were interacted with either the current treatment constant or the LAI/alternative oral ART constant. Covariates that were not statistically significant were removed for parsimony. Covariates of interest included: age (>40 years old), gender, race, sexual orientation, country, socioeconomic status, treatment challenge scenario, time since initiating ART, time since HIV diagnosis, stigma score, fear of needles, current medication adherence, current treatment satisfaction, changing therapy more than three times, and previously discussing switching with HCP.

## 3. Results

### 3.1. PLWH Characteristics

A total of 553 PLWH from the US (82%) and Canada (18%) completed the survey. The mean age was 39.3 years, and 66% of participants were male, 56% white/Caucasian, and 19% black/African American (Table 3). Overall, 62% of PLWH identified as heterosexual, while 29% PLWH identified as lesbian gay, bisexual, transgender, or queer (LGBTQ) and an additional 8% identified specifically as men having sex with men (MSM). The mean time since initial HIV diagnosis was reported as 9.4 years (Table 3), with over half of participants initiating treatment more than 3 years ago (54%). In total, 76% of PLWH (*n* = 421) saw their doctor ≥ 3 times a year, most commonly seeing infectious disease specialists (*n* = 230, 42%) and HIV specialists (*n* = 190, 34%) for management of their HIV. Overall, 70% of the PLWH sample (*n* = 389) self-reported high health literacy (average score of >2 on health literacy questions). Demographics and clinical characteristics were generally similar between US and Canadian patient participants, with some differences in the racial composition for each cohort (Table 3).

### 3.2. Physician Characteristics

In total, 456 physicians completed the survey (US 67%; Canada 33%) and reported having spent a mean of 16.1 years practicing medicine and a mean of 13.2 years specifically treating HIV (Table 4). Doctors spent a mean of 36% of their clinic time devoted to HIV care. Just over half of physicians (55%) identified as primary care/general practitioners. In the US, 39% identified themselves specifically as infectious disease specialists; in Canada, 17% identified as HIV specialists. Physician characteristics were otherwise generally similar between the US and Canada cohorts.

### 3.3. Current Treatment Satisfaction, Adherence and Stigma

PLWH and physicians reported a relatively high level of overall satisfaction (1 being totally unsatisfied, 7 being totally satisfied) with their current ART (mean scores = 5.5 and 5.2, respectively). However, only 28% (*n* = 155) of PLWH and 7% (*n* = 30) of physicians reported being “totally satisfied” with the current ART options. The most common reasons for dissatisfaction amongst PLWH (participants could choose more than one reason) were the requirement to take daily medication (*n* = 202, 37%), side-effects (*n* = 151, 27%), restrictions around when they can eat (*n* = 137, 25%), and the negative association of having a daily reminder of their HIV diagnosis (*n* = 133, 24%).

The majority of PLWH reported changing their HIV medication at least once in the past (*n* = 480, 88%), and 37% (*n* = 202) had changed their medication at least 3 times. In terms of current ART, more PLWH were taking a multiple tablet regimen (MTR) (*n* = 331, 56%) versus taking a single-tablet regimen (*n* = 242, 44%). Around half of PLWH reported forgetting to take medication in the past 4 weeks (*n* = 277, 50%), while around a quarter reported intentionally not taking medication in the past 4 weeks (*n* = 148, 27%). The most common reasons for intentionally not taking medication were social plans interfering with usual dosing time (*n* = 68, 12%) and work schedule interfering with usual dosing time (*n* = 71, 13%). For PLWH who forgot to take their medication, the mean number of days missed was 3.9 (SD: 4.2) out of 28 days.

PLWH were asked a series of questions to assess their perception of stigma associated with having HIV. Overall mean stigma score for PLWH was 20.2 (SD: 5.1), where 7 indicates lowest level of stigma and 28 indicates highest level of stigma. Only 6% (*n* = 32) of PLWH in the DCE population reported having not told anyone about their HIV diagnosis, with 35% (*n* = 196) having told their entire family and 16% (*n* = 86) having told all of their friends. However, 44% (*n* = 227) strongly agreed that they are very careful about who they reveal their HIV status to, 34% (*n* = 177) strongly agreed that they worried about people who know they have HIV telling others, and 34% (*n* = 175) strongly agreed that HIV hinders them from being intimate with other people.

PLWH were also asked how much they agreed or disagreed with various statements on the potential impact of the LAI ART on perceived social stigma and treatment satisfaction. More than 65% of the PLWH agreed with each of the following statements about potential impact of a LAI ART: it would put fewer restrictions on their life (*n* = 461, 83%), make them feel more in control of their life (*n* = 436, 79%), let them do more activities that they want to do (*n* = 419, 76%), remove the daily reminder of HIV (*n* = 413, 75%), make them feel more normal (*n* = 411, 74%), help them start a new chapter in their life (*n* = 407, 74%), reduce the chances that others would find out about their HIV status (*n* = 386, 70%), make them feel less ashamed/stigmatized (*n* = 373, 68%), and allow them to forget about their HIV (*n* = 365, 66%).

When PLWH were asked to select the scenario that most closely reflects their current treatment challenge (Table 2), about half selected either lifestyle (*n* = 218, 39%) or lifestyle and adherence (*n* = 70, 13%). The remaining PLWH selected side effects (*n* = 103, 19%), having no current challenges (*n* = 68, 12%), worry/anxiety/fatigue (*n* = 50; 9%), or disclosure (*n* = 44; 8%).

For physicians, the main reasons for recommending a change in ART treatment were resistance to current treatment (*n* = 320, 58%), toxicity/intolerance (*n* = 296, 54%), and virologic failure (*n* = 292, 53%). Overall, physicians reported that forgetting to take medication (*n* = 262, 58%) and side effects (*n* = 97, 21%) were the most common reasons for non-adherence reported by their patients. When asked which of three statements best describes their opinion of LAI therapies for the treatment of HIV in their practice, just over half of physicians (*n* = 248, 54%) selected “I am excited about the prospect of LAI treatment and see a clear place for them”, 43% (*n* = 196) selected “I would consider LAI treatments, but only under certain circumstances”, and the remaining 3% (*n* = 12) chose “I cannot see a place for LAI treatments”. Physicians also reported that they would offer or recommend a LAI to approximately half of their current PLWH (mean: 49%, SD: 30), if it were available.

### 3.4. Preference Estimates

**Figure 2:**
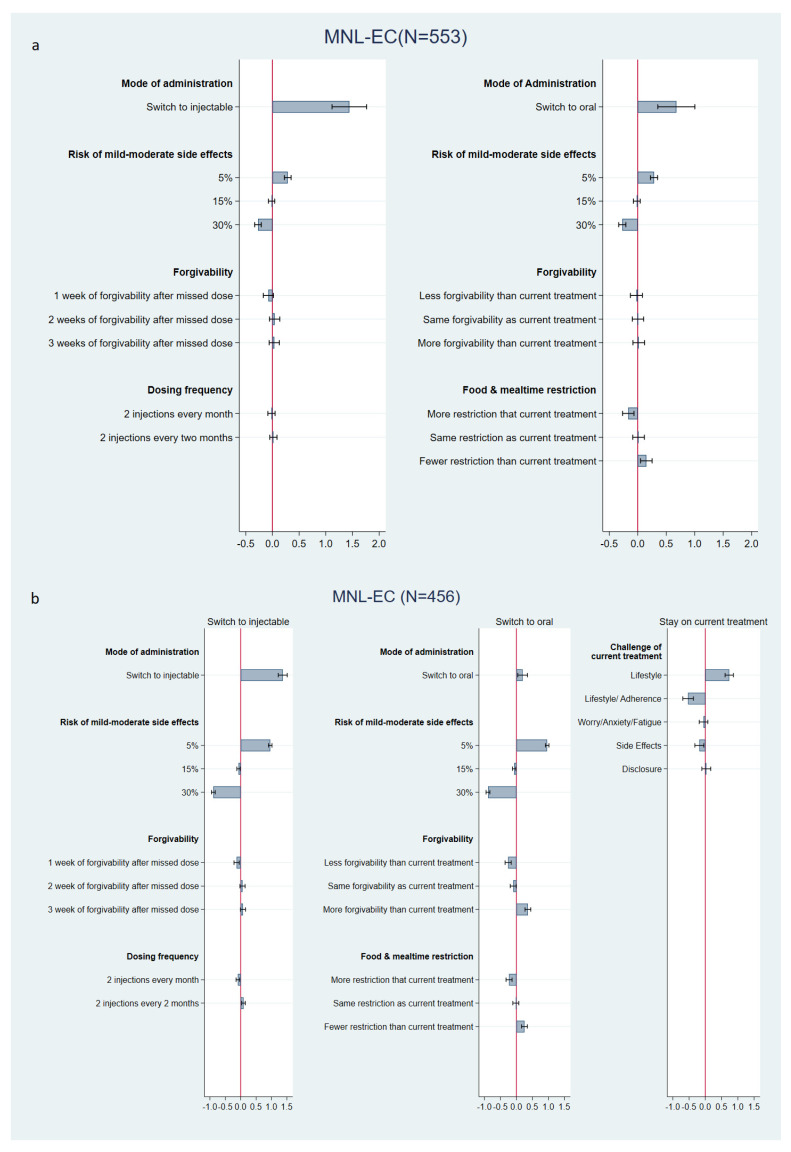
(**a**) Estimated marginal utilities: PLWH (N = 553). (**b**) Estimated marginal utilities: physicians (N = 456). PLWH—people living with HIV; SD—standard deviation; *** *p* < 0.001; PLWH: Final Log-Likelihood = −4817; Adjusted McFadden R^2^ = 0.338; Physicians: Final Log-Likelihood = −5788; Adjusted McFadden R^2^ = 0.276. In Figure 2a, bars to the right of the red line indicate a positive impact on the likelihood of switching to either injectable or a new oral, with longer bars indicating a stronger impact on preference. A statistically significant effect is present if the confidence interval does not overlap with 0. In Figure 2b, bars to the right of the red line indicate a positive impact on the likelihood of recommending a switch to either injectable or a new oral, with longer bars indicating a stronger impact. A statistically significant effect is present if the confidence interval does not overlap with 0.

DCE data quality indicators for PLWH and physicians were in line with similar studies in the literature (Table S1a,b), indicating that the participants were able to understand the DCE and were engaged [21]. The estimated marginal utilities from the DCE modelling are presented in Figure 2a,b (and Table S2a,b) for PLWH and physicians, respectively. Both PLWH and physician models had a good data fit. On average, PLWH and physicians had a significantly (*p* < 0.001) higher tendency to switch to a LAI than to switching to another oral ART or to remain on their current ART. PLWH and physicians also preferred treatments with a smaller risk of side effects (*p* < 0.001) and fewer food and mealtime restrictions (*p* < 0.01). While forgivability and dosing frequency did not significantly (*p* > 0.05) affect switching preferences of PLWH, physicians’ switching preferences were significantly affected by improvements in forgivability in oral ART (*p* < 0.001) or LAI (*p* < 0.01), with a preference for a two- or three-week LAI forgivability compared with one-week. In contrast to PLWH, physicians also had a significant preference for a LAI that is administered every two months instead of every month (*p* < 0.001).

Switching preferences were heterogeneous in both physician and PLWH populations, as indicated by the standard deviations in Figure 2a,b. Approximately 20% of PLWH (N = 106, 19%) made choices in the DCE indicating they were most likely to switch to a LAI, while being very reluctant to switch to an oral treatment (Figure S1a). These PLWH are expected to benefit most from a LAI, as their choices indicated a perception that LAI may overcome their current treatment challenges, while current oral ART may not offer a solution to these challenges. Among physicians, the vast majority displayed a choice tendency pattern to switch to either injectable or oral treatment (N = 413, 91%) (Figure S1b). Physicians’ tendency to recommend switching depended on the treatment challenges described to them (Table 2, Figure 2b). For example, they were significantly more likely (*p* < 0.001) to recommend staying on current treatment for PLWH who faced lifestyle problems without any adherence concerns and were more likely to recommend switching if PLWH had lifestyle concerns that affected adherence (*p* < 0.001) or if the PLWH struggled with side effects (*p* < 0.05).

To further understand preference heterogeneity among PLWH, socioeconomic and clinical characteristics were interacted with the constants of the model (Table S3). PLWH with a low income (<75,000 USD) were found to be more likely to prefer staying on their current treatment than PLWH with a high income (*p* < 0.001). Furthermore, PLWH who reported lifestyle challenges but no adherence concerns were likely to prefer staying on their current treatment compared with PLWH with more fundamental treatment challenges (*p* < 0.01). Heterosexual PLWH were more likely to switch to another oral medication than MSM or LGBTQ PLWH (*p* < 0.001). Heterosexual PLWH were also more likely than LGBTQ to switch to injectable (*p* < 0.05) with a similar but non-significant trend compared with MSM (*p* = NS). There were no significant differences among switching behaviors by racial group. Finally, the results suggest that fear of needles reduced the likelihood of PLWH switching to a LAI (*p* < 0.01), whereas self-reported non-adherence within the last four weeks increased the likelihood of PLWH switching to a LAI (*p* < 0.001).

### 3.5. Predicted Switching Probabilities

**Figure 3:**
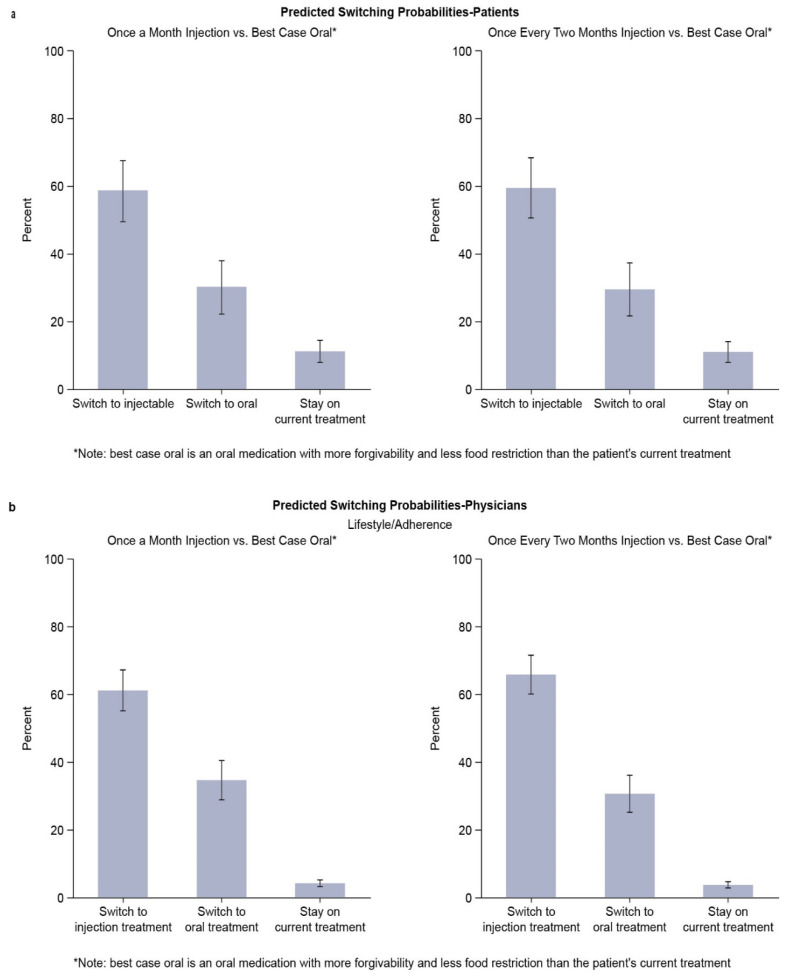
(**a**) Switching probabilities (best-case oral): PLWH. (**b**) Switching probabilities (best-case oral): physician; lifestyle and adherence challenge. PLWH—people living with HIV.

Using the parameter estimates from the DCE model, we estimated the predicted probabilities that patients and physicians would switch to LAI options when given the alternative choices of switch to the best-case oral treatment profile (i.e., more forgivable and fewer food restrictions than the current treatment) or staying on current treatment. Approximately 60% of PLWH were predicted to prefer switching to a LAI treatment (Figure 3a), regardless of LAI dosing frequency (monthly or every two months). Results were very similar among physicians, with a predicted uptake of the LAI ranging from 55–61% for monthly LAI and 60–66% for LAI every two months across all treatment challenges. Predicted uptake by physicians for the selected lifestyle and adherence treatment challenge is shown in Figure 3b.

Overall results from the DCE were directionally similar between the US and Canadian cohorts for both patients and physicians; however, some of the significant findings for the DCE in the combined US and Canada group and the US cohort were not significant in the Canadian sample, likely due to the smaller sample size.

## 4. Discussion

In this DCE preference survey, when participants were presented with choices to either remain on current oral therapy versus switching to LAI therapy administered either monthly or every two months, or an alternative oral therapy with different treatment attributes, choices of both patients and physicians indicated interest in switching to the LAI options. These findings are comparable with patient reported outcomes evaluated in the ATLAS (NCT02951052) and FLAIR (NCT02938520) clinical trials, where participants receiving a LAI administered monthly indicated high levels of preference for the LAI (98%) versus their prior oral therapy [12].

The survey data revealed that although the overall level of treatment satisfaction was high amongst PLWH and physicians, the majority of participants were not “fully satisfied” with current oral ART regimens, which suggests there is currently an opportunity to address unmet needs associated with existing ART treatment options. The main reasons for PLWH dissatisfaction were linked to the requirement for daily medication, treatment side-effects, the impact upon their personal lives brought about by restrictions around food/mealtimes, as well as the negative association of the daily reminder of living with HIV. A large proportion of PLWH indicated they were careful of who they told about their HIV diagnosis, and worrying about those who know telling others, indicating that PLWH feel that there is still a considerable amount of stigma regarding HIV diagnosis. These factors may contribute to patients’ preferences for ARTs, given that the use of a LAI ART would negate the need to take medication on a daily basis, therefore lessening the daily reminder of HIV, as well as removing the presence of ART medication bottles/packets, which may help to reduce feelings or fear of stigma amongst PLWH.

Additionally, although self-reported adherence was relatively good in the survey, which would be expected in patients who are virologically suppressed, over a quarter of PLWH still reported intentionally not taking their medication at times, often due to work and social interference. This indicates that there is still improvement needed in terms of maintaining consistent adherence to medication and scheduling daily dosing, which LAI ART may also help address.

The reasons PLWH cited for their dissatisfaction with treatment were good predictors of switching behavior in the DCE. For example, fewer side-effects and fewer food and meal restrictions were important factors for PLWH in influencing the decision to switch to either a LAI or an alternative oral. Conversely, neither frequency of injections (monthly or every two months) nor forgivability were important factors in the choices amongst PLWH, whereas for physicians, all of these factors were significantly valued in terms of treatment choices. This is in line with physician concerns about adherence of PLWH and factors that may influence adherence. Furthermore, PLWH with characteristics such as difficulties adhering to current dosing schedules were more likely to choose to switch to a LAI but not an alternative oral. These PLWH may benefit the most from the introduction of a LAI, because their preferences suggest they would not likely consider switching to another oral medication as a viable approach to overcoming their current treatment challenges. In a real-world setting, these findings could be especially relevant in helping to identify a subpopulation of PLWH in whom the introduction of a LAI could have a significant impact on improving the management of their HIV.

Other recent survey studies have identified a fairly consistent proportion of US PLWH interested in long-acting ART options (around 40–60%) [25,26,27]. A further US study (N = 263) assessing patient interest in switching to novel ART regimens found 39% of respondents were “very interested” in switching to an ART regimen of two injections every two months, 23% were “somewhat interested”, and 38% were not at all interested [28]. Differences in study design prevent direct comparison of the results to our current study; this other study provided respondents more choices of potential ART options, including a weekly pill and two implants every six months, which may have somewhat diluted the interest in LAI, in contrast to the current study offering only the option of a daily oral ART or a monthly or every two months LAI.

DCE results for the physicians indicated a high rate of recommending switching to LAI therapies (both in terms of their actual choices in the DCE and the predicted uptake). This could be in part due to the design of the current study, where physicians were asked to imagine that a PLWH had approached them with a particular treatment challenge and may have led physicians to recommend switching in the choice exercise more often than they might do in a normal clinical encounter. Nonetheless, in addition to the preference results from the DCE, over half the physicians (54.4%) explicitly responded that they were excited about the prospect of a LAI treatment option. An additional 43% indicated that they would consider the LAI under certain circumstances. Physicians specializing in internal medicine or family practice were the most inclined to recommend switching to LAI. It may be that this preference for switching could be due to certain physicians prioritizing patient treatment challenges differently.

It is important to note that many physicians indicated needing more information than was provided in the DCE to make their treatment decisions. The DCE is a simplified version of the real-world experience in terms of what physicians would encounter in their clinics when interacting with PLWH. Although some physicians participating in the qualitative pilot indicated an interest in more information about injection site reactions and the side effects of both the current and alternative treatment options, there was not highly consistent feedback on a specific area, and therefore, it was not feasible to add this information to the DCE. However, PLWH or physicians who were less likely to switch to a LAI or more inclined to switch to an alternative oral regimen may be a target of future research.

Whilst the overall results from the DCE indicate a high level of PLWH and physician preference for switching to a LAI, there are some limitations to this study to be considered. First, as previously noted, the scenarios presented in the DCE represent hypothetical decision making in a survey setting and may not reflect the actual choices that the participants would make or may not include all of the factors that a patient or physician would consider to make a treatment choice/recommendation. Attempts to minimize this potential effect included presentation of a realistic description of the LAI treatment option and careful initial selection of treatment attributes, which were then differentially tailored towards PLWH or physicians to reflect differences in treatment priorities.

Furthermore, recruitment for the survey was conducted via panels, and whilst this is an efficient means for conducting the study, as with all sampling approaches, the resulting samples may reflect some bias or may not be generalizable to the entire population of PLWH in US and Canada. In addition, it remains unknown if PLWH who chose not to participate had the same preferences as those who participated. Comparing the demographics of the PLWH population surveyed in this study with national statistics indicates some differences in demographic profiles [29,30]. The proportion of male PLWH in this study, 66% overall, was slightly lower than in reports of estimated prevalence across US and Canada (78% and 77%, respectively). In the US, the highest prevalence rate by race among PLWH is in Black/African Americans, followed by people of multiple races, and then Hispanic/Latino PLWH, whereas in the current study, there is a considerably larger proportion of White/Caucasian PLWH than Black/African Americans. The highest HIV transmission category reported in national statistics is men who have sex with men (74.5% of male infections were attributable to male-to-male sexual contact in the US, and 48.9% of HIV infections in Canada were estimated to be in men who have sex with men), while the largest sexual orientation category in this study was heterosexual (62.2%). This seemingly high proportion of heterosexuals could be at least partially due to the large proportion of female respondents (33%) in the study, many of whom may have identified as heterosexual. Lastly, the PLWH included in the survey self-reported their HIV diagnosis, viral suppression and length of time on therapy, which were not clinically verified. Despite these potential limitations, the large sample size increases the validity of our findings, as well as the likelihood of producing valuable information relevant to the HIV communities in the US and Canada.

## 5. Conclusions

This survey demonstrates a potentially strong interest for LAI ART options from both the PLWH and physician perspectives. On average neither PLWH nor physicians reported complete satisfaction with the currently available treatment options for HIV, and many of the issues reported with current daily oral ART regimens for some PLWH may be negated by switching to LAIs. The results of this study suggest that LAI therapy, with demonstrated non-inferiority against daily oral ART regimens in clinical trials in virologically suppressed patients, would be a welcome and valuable treatment option for both PLWH and the physicians who treat them.

## Figures and Tables

**Table 1:** DCE Attributes and Level.

Attributes	Attribute Description	Attribute Levels *(Variable Name)*
Dosing frequency	PLWH: How often you would receive the HIV treatment. For LAI, you would receive two injections each time at your HIV doctor’s clinic. Physicians: How often your PLWH would receive the HIV treatment. For LAI, two injections are given each time at your HIV clinic.	Oral: One pill, every day
		LAI: Two injections at the same visit every month ^ (*Frequency_Monthly*) Two injections at the same visit every two months (*Frequency_2 months*)
Risk of side effects	How many PLWH on a treatment develop mild to moderate bothersome side effects.	Oral and LAI: 5 out of 100 PLWH (5%) develops mild to moderate side effects. (*Risk_5*) 15 out of 100 PLWH (15%) develop mild to moderate side effects. (*Risk_15*) 30 out of 100 PLWH (30%) develop mild to moderate side effects.^ (*Risk_30*)
Forgivability	PLWH: The length of time that you have to take a missed dose without risking loss of viral suppression. You resume the normal dosing frequency after taking this catch-up dose. Physicians: the length of time PLWH have to take a missed dose without risking loss of viral suppression. PLWH resume the normal dosing frequency after taking this catch-up dose.	Oral: Less forgivability than the current treatment ^ (*Forgive_Less*) Same forgivability as the current treatment (*Forgive_Same*) More forgivability than the current treatment (*Forgive_More*)
		LAI: 1 week of forgivability after missed dose ^ (*Forgive_1 week*) 2 weeks of forgivability after missed dose (*Forgive_2 weeks*) 3 weeks of forgivability after missed dose (*Forgive_3 weeks*)
Food and mealtime restrictions	PLWH: How much you are restricted in what and when you eat. Physicians: How much PLWH are restricted in what and when they eat.	Oral: More restrictions than the current treatment ^ (*Restrict_More*) Same restrictions as the current treatment (*Restrict_Same*) Fewer restrictions than the current treatment (*Restrict_Fewer*)
		LAI: None

The Italics designates names of variables; ^—Reference category; DCE—discrete choice experiment; HIV—human immunodeficiency virus; LAI—long-acting injectable; PLWH—people living with HIV.

**Table 2:** Treatment challenge scenarios.

Challenge (*Variable Name*)	PLWH	Physician
Lifestyle (*Reference*)	You find that your current treatment increasingly interferes with your lifestyle. While the treatment is becoming an inconvenience, you generally manage to follow your treatment dosing schedule.	The treatment increasingly interferes with the PLWH’s busy lifestyle. However, the PLWH manages to adhere to your treatment advice.
Lifestyle and adherence (*Adhere*)	You notice that you are increasingly missing a treatment dose. You are concerned that the treatment does not fit very well into your lifestyle.	You notice that the PLWH increasingly misses a treatment dose. You are concerned that the treatment does not fit very well into your PLWH’s lifestyle.
Worry/Fatigue (*Worry*)	You worry about the need to take your HIV medicine daily and you are increasingly tired of having to take the medicine. Every time you take your HIV medicine it is a reminder of your HIV. Your treatment feels like a burden to you.	You notice that the daily need to take the medicine worries your PLWH and causes anxiety. You are concerned about how the PLWH will manage the treatment in the long term.
Side effects (*Side_effect*)	You are struggling with the side effects of your medication. The side effects are becoming increasingly burdensome and you wonder how you will be able to manage them in the future.	The PLWH struggles with the management of side effects. You are concerned about how the PLWH will manage the side effects in the future.
Disclosure (*Disclosue*)	You constantly worry that a friend, family member, or co-worker might discover your HIV medicine. Not many people know you have HIV and you carefully hide your pills.	The PLWH seems to constantly worry that friends, family members, or others discover the medicine. Not many people know the PLWH has HIV, and the PLWH is hiding the pills carefully.
No challenge (*Excluded for for physicians*)	You are generally satisfied with your current treatment. However, you may be curious to learn about new HIV treatments.	Not applicable in physician DCE

The Italics designates names of variables; DCE—discrete choice experiment; HIV—human immunodeficiency virus; LAI—long-acting injectable; PLWH—people living with HIV.

**Table 3:** Sociodemographic and clinical characteristics of PLWH.

	Overall (N = 553)	US (*n* = 453)	Canada (*n* = 100)
Gender, *n* (%)			
Female	183 (33.1)	146 (32.2)	37 (37.0)
Male	365 (66.0)	304 (67.1)	61 (61.0)
Transgender	5 (0.9)	3 (0.7)	2 (2.0)
Age, years			
Mean (SD)	39.3 (12.2)	39.5 (12.3)	38.4 (11.5)
Age group, *n* (%)			
>55 years old	82 (14.9)	72 (15.9)	10 (10.0)
Race ^a^, *n* (%)			
White/Caucasian	311 (56.2)	250 (55.2)	61 (61.0)
Black/African American	105 (19.0)	96 (21.2)	9 (9.0)
Hispanic/Latino	51 (9.2)	51 (11.3)	N/A
Asian	31 (5.6)	16 (3.5)	15 (15.0)
Other/Mixed race	54 (9.7)	40 (8.8)	14 (14.0)
Ethnicity, *n* (%)			
Hispanic/Latino	77 (13.9)	77 (17.0)	N/A
Not Hispanic/Latino	475 (85.9)	376 (83.0)	N/A
Sexual orientation, *n* (%)			
Straight	344 (62.2)	282 (62.3)	62 (62.0)
Lesbian, gay, bisexual, transgender, or queer (LGBTQ)	159 (28.8)	132 (29.1)	27 (27.0)
Men who have sex with men	46 (8.3)	35 (7.7)	11 (11.0)
Other	2 (0.4)	2 (0.4)	0
Prefer not to say	2 (0.4)	2 (0.4)	0
Income (USD), *n* (%)			
Less than USD 25,000	111 (20.1)	99 (21.9)	12 (12.0)
USD 25,000 to USD 49,999	82 (14.8)	67 (14.8)	15 (15.0)
USD 50,000 to USD 74,999	94 (17.0)	74 (16.3)	20 (20.0)
USD 75,000 to USD 99,999	97 (17.5)	86 (19.0)	11 (11.0)
USD 100,000 to USD 149,999	109 (19.7)	79 (17.4)	30 (30.0)
USD 150,000 or more	53 (9.6)	42 (9.3)	11 (11.0)
Prefer not to answer	7 (1.3)	6 (1.3)	1 (1.0)
Time since diagnosis (group), *n* (%)			
<1 year	14 (2.5)	13 (2.9)	1 (1.0)
1–2 years	103 (18.6)	84 (18.5)	19 (19.0)
2–5 years	142 (25.7)	109 (24.1)	33 (33.0)
5–10 years	118 (21.3)	98 (21.6)	20 (20.0)
>10 years	176 (31.8)	149 (32.9)	27 (27.0)
Time since diagnosis, years			
Mean (SD)	9.4 (8.8)	9.7 (9.0)	8.3 (8.2)
Time since initiating therapy, *n* (%)			
≤1 year ago	102 (18.4)	76 (16.8)	26 (26.0)
1 year to 3 years ago	152 (27.5)	123 (27.2)	29 (29.0)
3–5 years ago	78 (14.1)	64 (14.1)	14 (14.0)
5–10 years ago	96 (17.4)	86 (19.0)	10 (10.0)
10 years ago or longer	120 (21.7)	102 (22.5)	18 (18.0)
I do not remember	5 (0.9)	2 (0.4)	3 (3.0)

N/A—not applicable; SD—standard deviation; PLWH—people living with HIV; US—United States; ^a^—More than one racial category could be selected.

**Table 4:** Clinical experience of physicians.

	Overall (N = 456)	US (*n* = 305)	Canada (*n* = 151)
Time practicing medicine, years			
Mean (SD)	16.1 (8.4)	16.8 (8.4)	14.5 (8.1)
Median	15.0	16.0	13.0
Min–max	2.0–39.0	2.0–37.0	2.0–39.0
Time treating HIV			
Mean (SD)	13.2 (8.1)	14.6 (8.2)	10.5 (6.9)
Median	12.0	13.0	9.0
Min–max	2.0–34.0	2.0–34.0	2.0–30.0
% clinical time dedicated to HIV care			
Mean (SD)	35.7 (30.1)	37.8 (31.0)	31.3 (27.7)
Median	25.0	30.0	20.0
Min–max	1.0–100.0	1.0–100.0	1.0–90.0
Location, *n* (%)			
Rural/Countryside	29 (6.4)	22 (7.2)	7 (4.6)
Outskirts/Suburbs of a small city	38 (8.3)	27 (8.9)	11 (7.3)
Center or close to center of a small city	96 (21.1)	62 (20.3)	34 (22.5)
Outskirts/Suburbs of a large city	93 (20.4)	66 (21.6)	27 (17.9)
Center or close to center of a large city	200 (43.9)	128 (42)	72 (47.7)
Role, *n* (%)			
Infectious disease specialist	136 (29.8)	120 (39.3)	16 (10.6)
Internal medicine/primary care/general doctor/family practitioner	250 (54.8)	154 (50.5)	96 (63.6)
Physician assistant/nurse practitioner	13 (2.9)	5 (1.6)	8 (5.3)
HIV specialist	50 (11)	24 (7.9)	26 (17.2)
Immunologist	1 (0.2)	0	1 (0.7)
Other	6 (1.3)	2 (0.7)	4 (2.6)
Clinic/Facility, *n* (%)			
Closed system/integrated network	18 (3.9)	14 (4.6)	4 (2.6)
Large group practice	126 (27.6)	82 (26.9)	44 (29.1)
Small group or individual practice	145 (31.8)	99 (32.5)	46 (30.5)
Community or regional hospital	58 (12.7)	31 (10.2)	27 (17.9)
Ryan White clinic	19 (4.2)	19 (6.2)	0
Academic system or hospital	90 (19.7)	60 (19.7)	30 (19.9)

HIV—human immunodeficiency virus; SD—standard deviation; US—United States.

## Data Availability

The datasets generated and/or analyzed during the current study are available from the corresponding author on reasonable request.

## Supplementary Materials

The following supporting information can be downloaded at: https://www.mdpi.com/article/10.3390/jpm12030334/s1. Table S1: (a) Validity—PLWH; (b) Validity—Physicians; Table S2: (a) Multinomial Logistic Model (Effects Coding)—PLWH; (b) Multinomial Logistic Model (Effects Coding)—Physicians; Table S3: Mixed Logit Regression Model with Interaction Effects—PLWH; Figure S1: Distribution of Preferences for LAI and Oral Treatment (a) PLWH (b) Physicians.
