# Supplementary material for: Patient and Physician Preferences for Regimen Attributes for the Treatment of HIV in the United States and Canada
Source: J Pers Med. 2022 Feb 23;12(3):334. doi: 10.3390/jpm12030334 (PMC8948790; doi:10.3390/jpm12030334)
Supplement: Supplementary file 1 [file jpm-12-00334-s001.zip › Supplementary material.pdf]

Table S1a. Validity – PLWH

	Overall (N = 553)		US (N = 453)		Canada (N = 100)	
	N	%	N	%	N	%
**Repeat questions**						
Failed	156	28.2	119	26.3	37	37.0
Passed	397	71.8	334	73.7	63	63.0
**Trade offs**						
Always trading	376	68.0	303	66.9	73	73.0
Always switch to injection	84	15.2	70	15.5	14	14.0
Always switch to oral	46	8.3	43	9.5	3	3.0
Always choose current treatment	47	8.5	37	8.2	10	10.0
**Time to complete DCE survey (minutes)**						
5–10 minutes	72	13.0	56	12.4	16	16.0
10–15 minutes	144	26.0	120	26.5	24	24.0
15–20 minutes	107	19.3	85	18.8	22	22.0
20–25 minutes	85	15.4	78	17.2	7	7.0
25–30 minutes	48	8.7	39	8.6	9	9.0
More than 30 minutes	97	17.5	75	16.6	22	22.0

DCE = discrete choice experiment; PLWH = people living with HIV; US = United States.

Table S1b. Validity – Physicians

	Overall (N = 456)		US (N = 305)		Canada (N = 151)	
	N	%	N	%	N	%
**Repeat questions**						
Failed	137	30.0	86	28.2	51	33.8
Passed	319	70.0	219	71.8	100	66.2
**Trade offs**						
Always trading	434	95.2	287	94.1	147	97.4
Always recommend switch to injection	16	3.5	13	4.3	3	2.0
Always recommend switch to oral	2	0.4	1	0.3	1	0.7
Always recommend staying on current treatment	4	0.9	4	1.3	0	0.0
**Time to complete DCE survey (minutes)**						
5–10 minutes	48	10.5	31	10.2	17	11.3
10–15 minutes	93	20.4	66	21.6	27	17.9
15–20 minutes	63	13.8	43	14.1	20	13.2
20–25 minutes	55	12.1	36	11.8	19	12.6
25–30 minutes	37	8.1	23	7.5	14	9.3
More than 30 minutes	160	35.1	106	34.8	54	35.8

DCE = discrete choice experiment; US = United States.

Table S2a. Multinomial Logistic Model (Effects Coding) - PLHW

	Overall<br>MNL-EC (N = 553)<br>Marginal utilities<br>(SE)<br>[95% CI]	US<br>MNL-EC (N = 453)<br>Marginal utilities<br>(SE)<br>[95% CI]	Canada<br>MNL-EC (N = 100)<br>Marginal utilities<br>(SE)<br>[95% CI]
**Constants**			
Switch to Injectable Treatment	1.442***	1.411***	1.570***
	(0.165)	(0.188)	(0.340)
	[1.117–1.766]	[1.042–1.779]	[0.903–2.237]
Switch to Oral Treatment	0.679***	0.761***	0.322
	(0.166)	(0.190)	(0.344)
	[0.353–1.004]	[0.389–1.132]	[-0.353–0.997]
**Frequency (Injectable)**			
2 injections every month	-0.0181	-0.050	0.099
	(0.035)	(0.039)	(0.074)
	[-0.086–0.050]	[-0.127–0.027]	[-0.046–0.244]
2 injections every two months	0.018	0.050	-0.099
	(0.035)	(0.039)	(0.074)
	[-0.050–0.086]	[-0.027–0.127]	[-0.244–0.046]
**Risk of side effects**			
30 out of 100 patients (30%) develops mild to moderate side effects	-0.271***	-0.339***	-0.031
	(0.032)	(0.036)	(0.068)
	[-0.333– -0.209]	[-0.410– -0.268]	[-0.164–0.102]
15 out of 100 patients (15%) develops mild to moderate side effects	-0.016	0.001	-0.067
	(0.030)	(0.034)	(0.0665)
	[-0.075–0.044]	[-0.067–0.068]	[-0.197–0.064]
5 out of 100 patients (5%) develops mild to moderate side effects	0.287***	0.338***	0.098
	(0.032)	(0.036)	(0.070)
	[0.224–0.350]	[0.267–0.409]	[-0.038–0.234]
**Forgivability**			
Less forgivability as your current treatment (Oral)	-0.023	-0.019	-0.037
	(0.054)	(0.060)	(0.121)
	[-0.128–0.082]	[-0.136–0.099]	[-0.274–0.199]
Same forgivability as your current treatment (Oral)	0.005	0.014	-0.024
	(0.051)	(0.057)	(0.112)
	[-0.095–0.105]	[-0.098–0.126]	[-0.244–0.195]
More forgivability as your current treatment (Oral)	0.018	0.004	0.061
	(0.052)	(0.0586)	(0.116)
	[-0.084–0.120]	[-0.110–0.119]	[-0.165–0.289]
1 week of forgivability after missed dose (Injectable)	-0.076	-0.132*	0.112
	(0.049)	(0.055)	(0.103)
	[-0.171–0.019]	[-0.240– -0.023]	[-0.091–0.314]

	Overall<br>MNL-EC (N = 553)<br>Marginal utilities<br>(SE)<br>[95% CI]	US<br>MNL-EC (N = 453)<br>Marginal utilities<br>(SE)<br>[95% CI]	Canada<br>MNL-EC (N = 100)<br>Marginal utilities<br>(SE)<br>[95% CI]
2 weeks of forgivability after missed dose (Injectable)	0.041	0.063	-0.048
	(0.049)	(0.056)	(0.105)
	[-0.055–0.138]	[-0.046–0.172]	[-0.255–0.159]
3 weeks of forgivability after missed dose (Injectable)	0.035	0.069	-0.064
	(0.049)	(0.055)	(0.104)
	[-0.061–0.130]	[-0.039–0.177]	[-0.268–0.140]
**Food and mealtime restrictions**			
More restrictions than your current treatment	-0.166**	-0.209***	-0.018
	(0.051)	(0.058)	(0.113)
	[-0.267– -0.066]	[-0.322–-0.095]	[-0.240–0.204]
Same restrictions than your current treatment	0.014	0.024	-0.022
	(0.052)	(0.058)	(0.117)
	[-0.088– 0.116]	[-0.090–0.139]	[-0.251–0.206]
Fewer restrictions than your current treatment	0.152**	0.184**	0.040
	(0.051)	(0.059)	(0.114)
	[0.050–0.253]	[0.069–0.299]	[-0.183–0.263]
**Error component**	3.009***	3.114***	2.569***
	(0.120)	(0.137)	(0.246)
	[2.774–3.245]	[2.846–3.383]	[2.087–3.051]
**Log-likelihood**	-4817	-3839	-961
**McFadden Adjusted R2**	0.338	0.355	0.261

CI = confidence interval; MNL-EC = multinomial logit with an error component; PLWH = people living with HIV; SE = standard error; US = United States.

*** p<0.001, ** p<0.01, * p<0.05

Note: The coefficients for the reference levels of the categorical variables are calculated as the negative of the sum of the coefficients for the other levels.

Table S2b. Multinomial Logistic Model (Effects Coding) - Physician

	Overall<br>MNL-EC (N = 456)<br>Marginal utilities<br>(SE)<br>[95% CI]	US<br>MNL-EC (N = 305)<br>Marginal utilities<br>(SE)<br>[95% CI]	Canada<br>MNL-EC (N = 151)<br>Marginal utilities<br>(SE)<br>[95% CI]
**Constants**			
Switch to Injectable Treatment	1.367***	1.451***	1.248***
	(0.074)	(0.097)	(0.115)
	[1.222–1.513]	[1.261–1.641]	[1.023–1.474]
Switch to Oral Treatment	0.193*	0.139	0.292*
	(0.078)	(0.101)	(0.120)
	[0.041–0.345]	[-0.059–0.338]	[0.056–0.527]
**Stay on current treatment**			
Lifestyle	0.735***	0.804***	0.621***
	(0.065)	(0.081)	(0.108)
	[0.609–0.862]	[0.645–0.963]	[0.409–0.833]
Lifestyle & Adherence	-0.530***	-0.516***	-0.558***
	(0.083)	(0.104)	(0.140)
	[-0.693– -0.367]	[-0.720– -0.312]	[-0.832– -0.285]
Worry/Anxiety/Fatigue	-0.055	-0.095	0.025
	(0.068)	(0.086)	(0.112)
	[-0.188–0.078]	[-0.263–0.072]	[-0.195–0.244]
Side Effects	-0.182**	-0.231**	-0.108
	(0.071)	(0.089)	(0.117)
	[-0.320– -0.044]	[-0.405– -0.056]	[-0.337–0.121]
Disclosure	0.032	0.038	0.021
	(0.070)	(0.088)	(0.116)
	[-0.105–0.169]	[-0.134–0.210]	[-0.208–0.249]
**Frequency**			
Two injections every month	-0.092**	-0.147***	0.003
	(0.030)	(0.037)	(0.049)
	[-0.149– -0.034]	[-0.220– -0.074]	[-0.092–0.099]
Two injections every two months	0.092**	0.147***	-0.003
	(0.030)	(0.037)	(0.049)
	[0.034–0.149]	[0.074–0.220]	[-0.099–0.092]
**Risk of side effects**			
30 out of 100 patients (30%) develops mild to moderate side effects	-0.890***	-1.030***	-0.655***
	(0.031)	(0.040)	(0.049)
	[-0.950– -0.829]	[-1.109– -0.952]	[-0.750– -0.559]
15 out of 100 patients (15%) develops mild to moderate side effects	-0.071*	-0.064	-0.079
	(0.028)	(0.035)	(0.045)
	[-0.125– -0.017]	[-0.132–0.004]	[-0.168–0.010]
5 out of 100 patients (5%) develops mild to moderate side effects	0.961***	1.095***	0.734***

	Overall<br>MNL-EC (N = 456)<br>Marginal utilities<br>(SE)<br>[95% CI]	US<br>MNL-EC (N = 305)<br>Marginal utilities<br>(SE)<br>[95% CI]	Canada<br>MNL-EC (N = 151)<br>Marginal utilities<br>(SE)<br>[95% CI]
	(0.029)	(0.038)	(0.046)
	[0.904–1.017]	[1.021–1.168]	[0.643–0.824]
**Forgivability**			
1 week of forgivability after missed dose (Injectable)	-0.133**	-0.205***	-0.012
	(0.043)	(0.054)	(0.071)
	[-0.218– -0.049]	[-0.311– -0.099]	[-0.151–0.128]
2 weeks of forgivability after missed dose (Injectable)	0.059	0.097	0.001
	(0.043)	(0.055)	(0.071)
	[-0.026–0.144]	[-0.011–0.204]	[-0.139–0.140]
3 weeks of forgivability after missed dose (Injectable)	0.075	0.108*	0.011
	(0.042)	(0.053)	(0.069)
	[-0.007–0.156]	[0.004–0.212]	[-0.124–0.146]
Less forgivability as your current treatment (Oral)	-0.260***	-0.293***	-0.208**
	(0.048)	(0.061)	(0.079)
	[-0.354– -0.165]	[-0.414– -0.173]	[-0.362– -0.054]
Same forgivability as your current treatment (Oral)	-0.099*	-0.111	-0.074
	(0.049)	(0.062)	(0.079)
	[-0.194– -0.004]	[-0.232–0.011]	[-0.228–0.081]
More forgivability as your current treatment (Oral)	0.359***	0.404***	0.282***
	(0.046)	(0.059)	(0.076)
	[0.268–0.450]	[0.289–0.519]	[0.132–0.431]
**Food and mealtime restrictions**			
More restrictions than your current treatment	-0.231***	-0.279***	-0.154*
	(0.048)	(0.061)	(0.078)
	[-0.324– -0.137]	[-0.398– -0.161]	[-0.307– -0.001]
Same restrictions than your current treatment	-0.020	-0.018	-0.016
	(0.047)	(0.060)	(0.077)
	[-0.112–0.073]	[-0.135–0.099]	[-0.168–0.135]
Fewer restrictions than your current treatment	0.250***	0.297***	0.170*
	(0.046)	(0.059)	(0.077)
	[0.159–0.341]	[0.183–0.412]	[0.020–0.320]
**Error component**	1.307***	1.407***	1.134***
	(0.053)	(0.070)	(0.080)
	[1.204–1.411]	[1.271–1.544]	[0.977–1.291]
**Log-likelihood**	-5789	-3708	-2049
**McFadden Adjusted R2**	0.276	0.305	0.222

CI = confidence interval; MNL-EC = multinomial logit with an error component; SE = standard error; US = United States.

*** p<0.001, ** p<0.01, * p<0.05

Note: The coefficients for the reference levels of the categorical variables are calculated as the negative of the sum of the coefficients for the other levels.

Table S3. Mixed Logit Regression Model with Interaction Effects - PLWH

	Overall (N = 553) MXL	
	Marginal utilities (SE)	95% CI
**Constants**		
Switch to LAI Treatment: mean	1.749***	[1.385–2.112]
	(0.185)	
Switch to LAI Treatment: standard deviation	2.732***	[2.450–3.015]
	(0.144)	
Switch to Oral Treatment: mean	0.935***	[0.583–1.287]
	(0.180)	
Switch to LAI Treatment: standard deviation	2.438***	[2.172–2.704]
	(0.136)	
**Interaction Effects**		
Switch to LAI *Non- Straight	-0.034	[-0.324–0.256]
	(0.148)	
Switch to LAI * Straight	0.034	[-0.256–0.324]
	(0.148)	
Switch to oral * Non-Straight	-0.710***	[-0.987– -0.433]
	(0.141)	
Switch to oral * Straight	0.710***	[0.433–0.987]
	(0.141)	
Switch to LAI * Do not fear needles	0.395**	[0.096–0.694]
	(0.153)	
Switch to LAI * Fear needles	-0.395**	[-0.694– -0.096]
	(0.153)	
Switch to oral * Do not fear needles	-0.243	[-0.521–0.035]
	(0.142)	
Switch to oral * Fear needles	0.243	[-0.035–0.521]
	(0.142)	
Switch to LAI * Did not forget medicine in the past 4 weeks	-0.731***	[-1.013– -0.449]
	(0.144)	

	Overall (N = 553) MXL	
	Marginal utilities (SE)	95% CI
Switch to LAI * Forget medicine in the past 4 weeks	0.731***	[0.449–1.013]
	(0.144)	
Switch to oral * Did not forget medicine in the past 4 weeks	-0.169	[-0.435–0.097]
	(0.136)	
Switch to oral * Forget medicine in the past 4 weeks	0.169	[-0.097–0.435]
	(0.136)	
Switch to LAI * Low stigma	-0.240	[-0.534–0.054]
	(0.150)	
Switch to LAI * High stigma	0.240	[-0.054–0.534]
	(0.150)	
Switch to oral * Low stigma	-0.475***	[-0.752– -0.199]
	(0.141)	
Switch to oral * High stigma	0.475***	[0.199–0.752]
	(0.141)	
Current treatment * Income below 75k	0.591***	[0.376–0.806]
	(0.110)	
Current treatment * Income above 75k	-0.591***	[-0.806– -0.376]
	(0.110)	
Current treatment * Less than 10 years since diagnosis	-0.430***	[-0.661– -0.199]
	(0.118)	
Current treatment * More than 10 years since diagnosis	0.430***	[0.199–0.661]
	(0.118)	
Current treatment * No Challenge	-0.227	[-0.760–0.307]
	(0.272)	
Current treatment * Lifestyle	0.573**	[0.206–0.940]
	(0.187)	
Current treatment * Adherence	-0.073	[-0.599–-0.454]
	(0.268)	
Current treatment * Worry/Anxiety/Fatigue	-0.084	[-0.689–-0.522]
	(0.309)	
Current treatment * Side Effects	-0.154	[-0.615–-0.308]

	Overall (N = 553) MXL	
	Marginal utilities (SE)	95% CI
	(0.235)	
Current treatment * Disclosure	-0.036	[-0.673–0.601]
	(0.325)	
**Frequency**		
2 injections every month (LAI)	-0.018	[-0.086–0.049]
	(0.034)	
2 injections every two months (LAI)	0.018	[-0.049–0.086]
	(0.034)	
**Risk of side effects**	-0.022***	[-0.026– -0.017]
	(0.002)	
**Forgivability**		
1 week of forgivability after missed dose (LAI)	-0.078	[-0.172–0.017]
	(0.048)	
2 weeks of forgivability after missed dose (LAI)	0.041	[-0.055–0.136]
	(0.049)	
3 weeks of forgivability after missed dose (LAI)	0.037	[-0.058–0.132]
	(0.048)	
Less forgivability as your current treatment (oral)	-0.017	[-0.121–0.088]
	(0.053)	
Same forgivability as your current treatment (oral)	0.004	[-0.096–0.103]
	(0.051)	
More forgivability as your current treatment (oral)	0.013	[-0.089–0.115]
	(0.052)	
**Food and mealtime restrictions**		
More restrictions than your current treatment	-0.168***	[-0.268– -0.068]
	(0.051)	
Same restrictions than your current treatment	0.017	[-0.085–0.119]
	(0.052)	
Fewer restrictions than your current treatment	0.151**	[0.050–0.253]
	(0.052)	
**Log-likelihood**	-4708	

	Overall (N = 553) MXL	
	Marginal utilities (SE)	95% CI
**BIC**	9654	
**McFadden Adjusted R2**	0.351	

BIC = Bayesian information criterion; CI = confidence interval; LAI = long-acting injectable; MXL = mixed logit model; PLWH = people living with HIV; SE = standard error.

*** p<0.001, ** p<0.01, * p<0.05

Note: The coefficients for the reference levels of the categorical variables are calculated as the negative of the sum of the coefficients for the other levels.

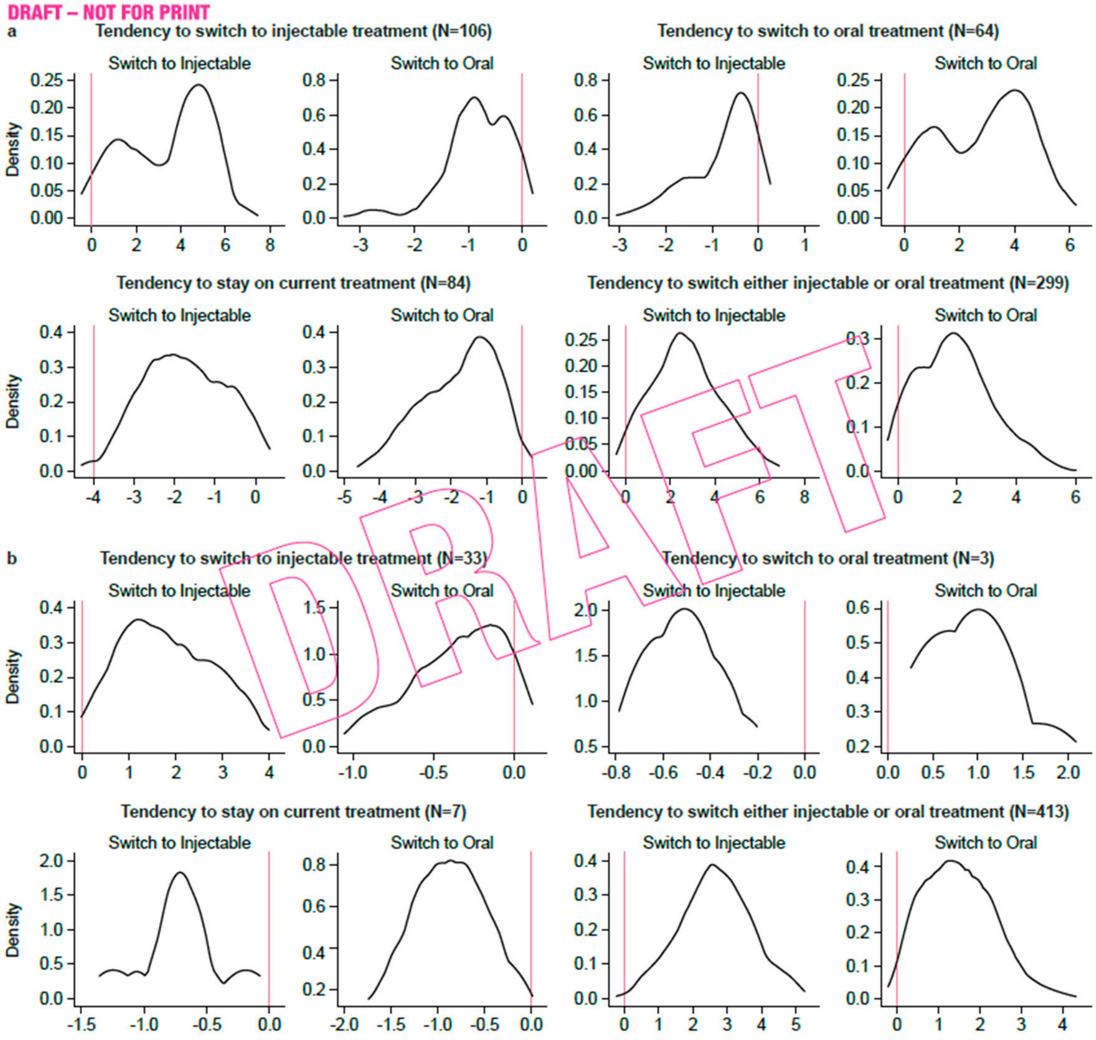

Figure S1. Distribution of Preferences for LAI and Oral Treatment a) PLWH[a] b) Physician[b]

PLWH = people living with HIV; [a]This density plot figure shows the four different groups defined based on the PLWH's tendency to switch treatment. The tendency to switch was obtained for each respondent as the estimated individual specific alternative specific constant. The distribution to the right of the line indicates a positive preference for switching treatment; [b]This density plot figure shows the four different groups defined based on the physician's tendency to recommend switching. The tendency to switch was obtained for each respondent as the estimated individual specific alternative specific constant. The distribution to the right of the line indicates a positive preference for switching treatment.
